# Action-Aware Multimodal Wavelet Fusion Network for Quantitative Elbow Motor Function Assessment Using sEMG and Robotic Kinematics

**DOI:** 10.3390/s26030804

**Published:** 2026-01-25

**Authors:** Zilong Song, Pei Zhu, Cuiwei Yang, Daomiao Wang, Jialiang Song, Daoyu Wang, Fanfu Fang, Yixi Wang

**Affiliations:** 1 Department of Biomedical Engineering, College of Biomedical Engineering, Fudan University, Shanghai 200433, China; zlsong23@m.fudan.edu.cn (Z.S.); wangdm23@m.fudan.edu.cn (D.W.); 2 Changhai Hospital, Naval Medical University, Shanghai 200433, China; 17321117846@163.com (P.Z.); songjl0906@163.com (J.S.); 3 Zdroid Medtech Co., Ltd., Shanghai 201210, China; dywang@zdmedtech.com (D.W.); yxwang@zdmedtech.com (Y.W.)

**Keywords:** continuous wavelet transform, multimodal fusion, sEMG, robotic-assisted rehabilitation, quantitative assessment

## Abstract

Accurate upper-limb motor assessment is critical for post-stroke rehabilitation but relies on subjective clinical scales. This study proposes the Action-Aware Multimodal Wavelet Fusion Network (AMWFNet), integrating surface electromyography (sEMG) and robotic kinematics for automated Fugl-Meyer Assessment (FMA-UE)-aligned quantification. Continuous Wavelet Transform (CWT) converts heterogeneous signals into unified time-frequency scalograms. A learnable modality gating mechanism dynamically weights physiological and kinematic features, while action embeddings encode task contexts across 18 standardized reaching tasks. Validated on 40 participants (20 post-stroke, 20 healthy), AMWFNet achieved 94.68% accuracy in six-class classification, outperforming baselines by 9.17% (Random Forest: 85.51%, SVM: 85.30%, 1D-CNN: 91.21%). The lightweight architecture (1.27 M parameters, 922 ms inference) enables real-time assessment-training integration in rehabilitation robots, providing an objective, efficient solution.

## 1. Introduction

Stroke remains a leading cause of long-term disability worldwide, and approximately 50–70% of survivors continue to experience upper-limb motor impairments that significantly restrict activities of daily living [1,2,3]. Accurate and efficient assessment of upper-limb motor function is therefore crucial for designing personalized rehabilitation plans and monitoring recovery. However, current clinical practice still relies heavily on subjective rating scales, such as the Fugl–Meyer Assessment for Upper-Limb (FMA-UE) [4], Brunnstrom Recovery Stages [5], Action Research Arm Test (ARAT), Wolf Motor Function Test (WMFT) [6], and Modified Ashworth Scale (MAS) [7], which are time-consuming, susceptible to inter-rater variability, and insufficiently sensitive to subtle functional changes [8].

To address these limitations, surface electromyography (sEMG) has emerged as a powerful tool for objective motor assessment. As a non-invasive biomarker of neuromuscular activity, sEMG provides direct insight into neural drive, muscle recruitment patterns, and electrophysiological dynamics [9]. Previous studies have successfully applied sEMG to quantify muscle fatigue [10], identify pathological synergies [11], and differentiate between spasticity and voluntary contraction in stroke patients [12]. While sEMG serves as a proxy for neural drive and muscle activation intensity, it does not directly quantify the external mechanical force. The relationship between sEMG amplitude and muscle force is non-linear and heavily influenced by joint angles, muscle fatigue, and contraction dynamics [13,14].

Parallel to physiological monitoring, robotic-assisted rehabilitation systems have gained prominence by delivering intensive, task-oriented therapy while alleviating therapist workload [15]. Importantly, these robots function as high-fidelity data acquisition platforms, capable of recording multi-dimensional kinematic parameters—including velocity, acceleration, and jerk (movement smoothness)—with temporal precision unattainable through manual observation [16]. This dual functionality enables “assessment-training integration,” wherein the robot simultaneously administers therapy and quantitatively evaluates motor performance in real time. However, existing robotic systems lack the intelligence to autonomously map raw sensor data to clinically meaningful functional scores, limiting their utility for automated assessment.

Despite the availability of sEMG and robotic sensing technologies, a critical gap persists between data acquisition and clinical decision-making [17]. While sEMG captures neural intent and kinematics quantify executed motion, no integrated framework exists to synergistically fuse these complementary modalities for automated upper-limb functional assessment. Existing attempts have predominantly relied on single-modality analysis or naive feature concatenation, neglecting the complex, task-dependent interactions between neuromuscular signals and movement execution [18,19]. Furthermore, the influence of action-specific variables (e.g., movement direction and amplitude) on multimodal assessment remains underexplored, limiting both the granularity and clinical applicability of current methods.

To bridge this gap, we propose the Action-Aware Multimodal Wavelet Fusion Network (AMWFNet). This deep learning framework integrates Continuous Wavelet Transform (CWT)-based signal representation with dynamic modality fusion to automate the assessment process. The framework operates on a clinically structured protocol comprising nine standardized planar reaching tasks (spanning three directions and three amplitudes). Crucially, these kinematic tasks are explicitly mapped to the FMA-UE Elbow Flexion and Extension sub-scores, treating the assessment as a refined classification problem. Three key innovations distinguish AMWFNet from existing approaches: (1) CWT-based feature extraction preserves non-stationary dynamics of signals in time-frequency space; (2) learnable modality gating dynamically adjusts the contribution of sEMG and kinematic channels based on signal reliability; and (3) action embedding explicitly encodes task-specific context to enhance classification granularity.

The core objectives of this study are threefold: (1) to develop a standardized, end-to-end deep learning framework that automates functional assessment, reducing clinician subjectivity and enhancing efficiency; (2) to validate the performance gains achieved through synergistic fusion of multimodal physiological and kinematic data with explicit action-context modeling; and (3) to demonstrate the feasibility of real-time “assessment-training integration” in robotic rehabilitation systems.

Our contributions are summarized as follows:

1. A novel multimodal fusion architecture that leverages learnable gating to dynamically weight sEMG and kinematic inputs, achieving superior performance over fixed-weight concatenation.
2. A clinically aligned assessment protocol that maps 18 task variations to specific FMA-UE Elbow Flexion and Extension sub-scores, ensuring generalizability across diverse functional contexts.
3. Extensive validation on 40 participants (20 stroke, 20 healthy) demonstrating 94.68% six-class classification accuracy, with ablation studies confirming the necessity of each architectural component.
4. Clinically interpretable insights through attention weight visualization, revealing task-specific muscle recruitment patterns aligned with known pathophysiology.

## 2. Materials and Methods

### 2.1. Study Overview and Workflow

**Figure 1:**
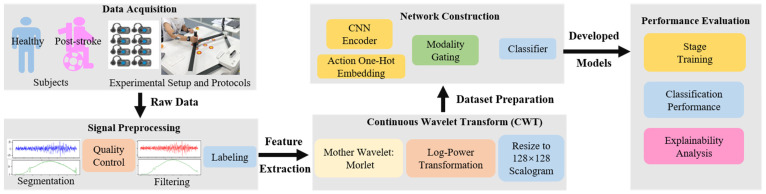
Schematic overview of the proposed experimental and methodological framework.

As illustrated in Figure 1, the study workflow comprises four sequential stages. First, in Data Acquisition, synchronous sEMG and kinematic data are collected from healthy and post-stroke subjects during 18 standardized robot-guided elbow flexion-extension tasks. Second, Signal Preprocessing entails segmentation, quality control, filtering, and labeling. This leads to CWT Feature Extraction, where signals are transformed into 128 × 128 scalograms using the Morlet wavelet to capture temporo-spectral dynamics. Third, Network Construction establishes the AMWFNet architecture, integrating a CNN encoder, action one-hot embedding, modality gating, and a classifier. Finally, Performance Evaluation implements a two-stage training strategy (pre-training on robot-guided passive data and fine-tuning on active voluntary data) and assesses the model via standard classification metrics and explainability analysis.

### 2.2. Participants and Data Acquisition

A total of 40 participants were recruited from Shanghai Changhai Hospital, comprising 20 post-stroke survivors with chronic hemiplegia and 20 healthy controls (see Table 1 for detailed demographic characteristics). The inclusion criteria for the stroke group required an FMA-UE score between 20 and 50. This range was selected to ensure participants possessed sufficient residual motor function to engage in the experimental tasks while exhibiting quantifiable motor impairments. The healthy control group was recruited to establish a baseline for normative motor performance.

This study was conducted in compliance with the Declaration of Helsinki and was approved by the Ethics Committee of Shanghai Changhai Hospital (Approval No. CHEC2023-290). Written informed consent was obtained from all individuals prior to participation.

**Figure 2:**
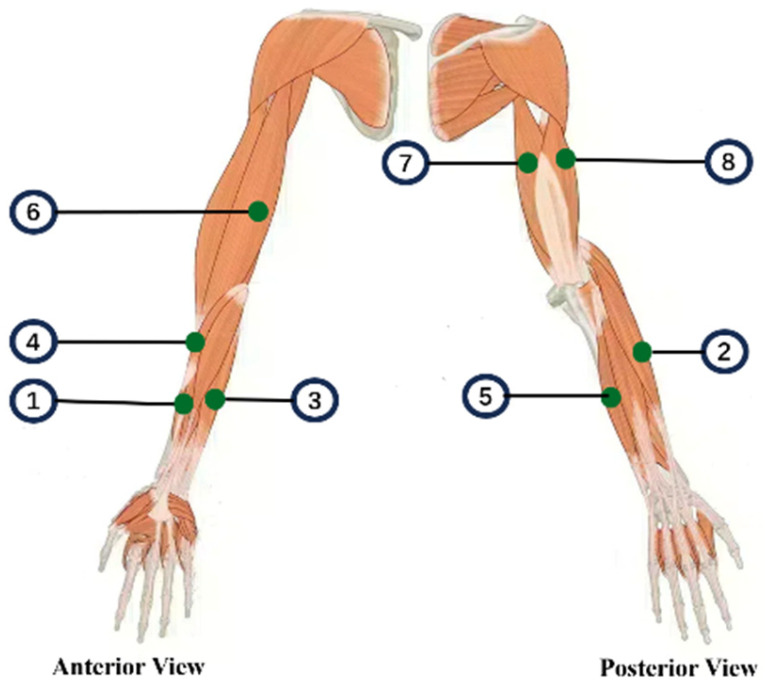
Schematic representation of the electrode placement sites. The corresponding muscles are: ① Flexor carpi radialis (FCR), ② Extensor carpi radialis (ECR), ③ Flexor carpi ulnaris(FCU), ④ Brachioradialis (BR), ⑤ Extensor carpi ulnaris (ECU), ⑥ Biceps brachii (BB), ⑦ Triceps brachii long head (TBL), and ⑧ Triceps brachii lateral head (TBLa).

Synchronous multimodal data were acquired using an integrated sensing framework. To comprehensively monitor neuromuscular activity, surface electromyography (sEMG) signals were recorded from eight muscles governing elbow and wrist movements, as illustrated in Figure 2. The target muscles included the flexor carpi radialis (FCR), extensor carpi radialis (ECR), flexor carpi ulnaris (FCU), brachioradialis (BR), extensor carpi ulnaris (ECU), biceps brachii (BB), and the triceps brachii long (TBL) and lateral heads (TBLa). This selection encompassed the primary agonist, antagonist, and synergist muscles involved in the target tasks. sEMG signals were captured using the BTS FREEEMG 300 wireless system (BTS Bioengineering, Milan, Italy) at a sampling rate of 1 kHz.

**Figure 3:**
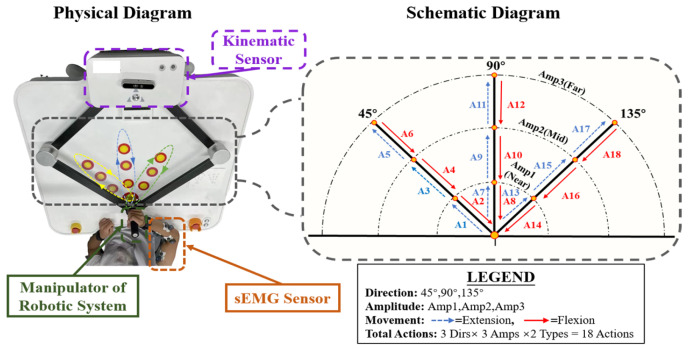
Experimental setup and schematic representation of the robot-assisted movement tasks. Physical robotic setup with multimodal sensors (**left**) and corresponding planar reaching trajectories at 45°, 90°, and 135° (**right**).

Concurrently, kinematic data of the robot’s end-effector were recorded via the built-in sensors of the rehabilitation robot ArmGuider (Zdroid Medtech, Shanghai, China) (Figure 3) at a sampling rate of 500 Hz. The primary recorded metric was velocity; acceleration and jerk were subsequently derived via numerical differentiation to quantify movement dynamics and control smoothness.

To capture a comprehensive profile of motor function and account for the influence of movement direction and amplitude, we designed a structured experimental protocol based on nine planar spatial targets. As illustrated in Figure 3, these targets are systematically defined by the intersection of three movement orientations (45°, 90° and 135°) and three radial distances (Near, Mid, and Far). However, to fully capture the dynamic characteristics of upper-limb movement, the interaction with each spatial target was decomposed into two distinct motion phases: Reaching Out (Extension) and Returning (Flexion). Consequently, this design yields a total of 18 distinct movement actions (9 spatial targets × 2 motion types).

The actions are indexed sequentially (Action IDs 1–18) following a “Direction-Major, Distance-Minor” numbering rule. Specifically, the three directions—45°, 90° and 135°—correspond to Action IDs 1–6, 7–12, and 13–18, respectively. Within each direction, targets are processed from near to far; for each target, the odd-numbered ID represents the Reaching Out phase, while the subsequent even-numbered ID represents the Returning phase.

To ensure clinical validity, the FMA-UE score for each participant was independently assessed by three experienced rehabilitation therapists, with the final score determined by the consensus of at least two raters. The classification labels (Score 0, 1, 2) were defined based on the specific sub-items of the FMA-UE scale: Score 0 indicates no voluntary movement or severe impairment; Score 1 denotes partial movement or movement performed with synergistic compensation; and Score 2 represents normal movement execution. Since the stroke cohort in this study primarily consisted of patients with moderate-to-severe impairments (Scores 0 and 1), data from the healthy control group were systematically assigned to Score 2 [20,21]. This assignment aligns with the clinical definition of the FMA, where healthy motor function constitutes the upper bound of the assessment scale. Consequently, the model is designed to map input features to a functional continuum from severe impairment (Score 0) to normal motor control (Score 2).

**Figure 4:**
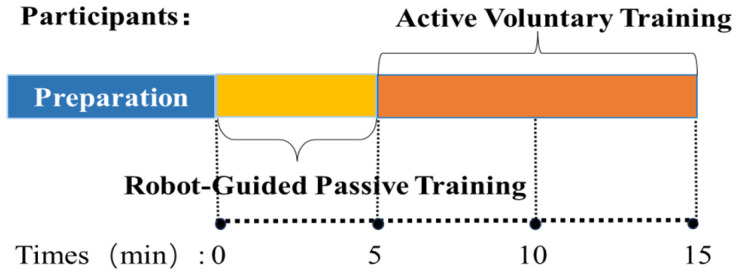
Schematic illustration of the data acquisition phases and their respective durations.

Following a preparation phase (Figure 4), the protocol proceeded with 5 min of robot-guided passive training for familiarization. This was followed by 10 min of active voluntary training, where participants performed the target reaching movements with maximal voluntary effort. To guarantee precise temporal alignment between the heterogeneous sensor streams, a hardware-triggered synchronization mechanism was implemented. A shared external trigger signal was utilized to simultaneously initiate data acquisition on both the sEMG system and the robot prior to each trial. This approach eliminated temporal drift and ensured the integrity of the multimodal data fusion.

### 2.3. Signal Preprocessing and CWT Feature Extraction

#### 2.3.1. Data Segmentation and Labeling

Continuous multimodal streams were segmented into discrete trials corresponding to the 18 specific elbow flexion and extension movements. To facilitate supervised learning, each trial was labeled with the specific action identifier and the subject’s FMA-UE elbow flexion and extension sub-scores, discretized into three impairment levels (0, 1, 2) [22,23]. This labeling strategy ensures every sample is explicitly associated with its clinical context and task difficulty.

#### 2.3.2. sEMG Preprocessing

To isolate relevant physiological signals, the 8-channel sEMG data underwent a two-stage zero-phase filtering cascade. First, a 50 Hz notch filter (second-order Butterworth, Q = 30) was applied to suppress power-line interference. Subsequently, a fourth-order Butterworth band-pass filter (20–450 Hz) was employed to retain motor unit action potentials while attenuating motion artifacts and baseline drift [24,25]. Zero-phase implementation was strictly applied to prevent temporal distortion, thereby preserving signal integrity for subsequent time-frequency analysis [26,27].

#### 2.3.3. Kinematic Preprocessing

Raw velocity data were differentiated to yield acceleration and jerk, providing a comprehensive view of motor dynamics. To suppress high-frequency quantization noise, a two-stage smoothing pipeline was applied to all kinematic channels: a zero-phase 4th-order Butterworth low-pass filter (cutoff: 20 Hz) followed by a 15 ms Gaussian-weighted moving average [28].

#### 2.3.4. Continuous Wavelet Transform (CWT) Implementation

We employed CWT to convert preprocessed 1D signals into 2D time-frequency scalograms, capturing the non-stationary characteristics of multimodal signals. The complex Morlet wavelet ($\omega_{0}=6$) was selected for its capacity to capture recruitment phase information and its biophysical resemblance to action potentials. Empirical validation on pilot data (n=5) demonstrated that Morlet yielded 5.3% and 4.1% higher class-separability (Fisher’s discriminant ratio) compared to Daubechies (db4) and Symlet (sym8) wavelets, respectively [29,30]. Scales were tailored to modality-specific spectral characteristics: 128 scales spanning 20–450 Hz for sEMG, and 0.5–50 Hz for kinematics [31]. These settings were finalized after an empirical grid search on a validation subset, where increasing scales beyond 128 yielded no significant performance gain but increased computational cost [32].

The CWT coefficients were processed through a standardized pipeline: (1) power spectrum conversion (${\left|W(a,b)\right|}^{2}$); (2) log1p transformation for dynamic range compression; (3) resizing to 128×128 via bilinear interpolation; and (4) [0, 1] normalization. This yielded a final input tensor of 11 scalograms (8 sEMG, 3 Kinematics) per trial for the AMWFNet.

### 2.4. Action-Aware Multimodal Wavelet Fusion Network (AMWFNet)

#### 2.4.1. Multimodal Image Encoder

**Figure 5:**
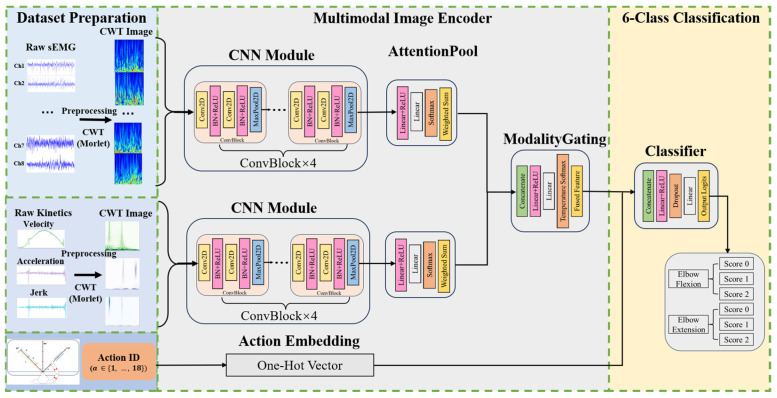
Schematic architecture of the proposed Action-Aware Multimodal Wavelet Fusion Network (AMWFNet).

To extract hierarchical features from the time-frequency representations, we designed a structurally identical CNN-based ImageEncoder for both sEMG and kinematic branches. The encoder processes a stack of CWT scalograms—8 channels for sEMG and 3 channels for kinematics—mapping them into a high-dimensional feature space (Figure 5).

The feature extraction module is composed of four stacked ConvBlocks. Inside each block, we employ a double-convolution design: two 3×3 convolutional layers, each followed by Batch Normalization and ReLU activation, concluding with a 2×2 Max Pooling layer for spatial downsampling.

As the spatial resolution decreases, the number of feature channels progressively doubles, following a sequence of 1→32→64→128→128. At the final stage, a Global Average Pooling (GAP) layer compresses the spatial domain (H×W→1×1). Consequently, the encoder transforms the input batch into a feature tensor $F\in R^{N\times D}$, preserving the feature dimension (N=128) for the subsequent attention mechanism [33].

#### 2.4.2. Channel-Wise Attention Pooling

Different muscles and kinematic indicators contribute unevenly to the assessment of motor function. To address this, we implemented a Channel-wise Attention Pooling module. For an input feature tensor $F\in R^{N\times D}$, the module computes a scalar attention weight $\alpha_{i}$ for each channel $\alpha_{i}$ [34].

Specifically, the attention mechanism is parameterized by a lightweight Multilayer Perceptron (MLP). This MLP consists of a projection layer reducing the feature dimension from D to $\frac{D}{2}$ (i.e., 128→64), followed by a ReLU activation and a final linear layer outputting a scalar score. These scores are normalized via a Softmax function across the N channels [31]. The final modality-specific feature vector $f_{modality}$ is obtained by the weighted sum of the channel features:

$$f_{modality}=\sum_{i=1}^{N}\alpha_{i}\cdot F_{i}$$

This mechanism enables the network to dynamically emphasize informative channels—such as the agonist Biceps Brachii during flexion—while suppressing irrelevant signals or noise. To visualize the underlying decision-making logic, we extracted and aggregated attention weight vectors to test the hypothesis that the network prioritizes muscles associated with pathological synergies and prime movers, while dynamically shifting kinematic focus between velocity and acceleration according to task-specific force requirements.

#### 2.4.3. Modality Gating Fusion Mechanism

To fuse the features from the sEMG ($f_{emg}$) and kinematic ($f_{kine}$) branches, we employ a learnable Modality Gating mechanism rather than naive concatenation. This module dynamically assigns importance weights to each modality based on their inherent feature content [35].

The two feature vectors are first concatenated to form a joint representation $f_{cat}\in R^{2D}$. A gating MLP, comprising a hidden layer of size 256 with ReLU activation, maps to a 2-dimensional logit vector $l=[l_{emg},l_{kine}]$. The final fusion weights w are computed using a temperature-scaled Softmax function [36,37]:

$$w_{m}=\frac{\exp(l_{m}/\tau )}{\sum_{k\in \{emg,kine\}}\exp(l_{k}/\tau )}$$

where τ is the temperature parameter. In our framework, τ regulates the sharpness of the weight distribution; a lower τ encourages a “winner-takes-all” selection of the most reliable modality, whereas a higher τ promotes a softer, more balanced fusion. The final fused vector is obtained via:

$$f_{fused}=w_{emg}\cdot f_{emg}+w_{kine}\cdot f_{kine}$$

The proposed dynamic gating mechanism automatically optimizes the weighting ratio between physiological (sEMG) and behavioral (kinematic) data, prioritizing the most informative modality for each specific sample.

#### 2.4.4. Action-Aware Classification Head

The fused feature vector $f_{fused}$ is augmented with task-specific context. To maintain parameter efficiency while preserving label distinctiveness, we utilize a direct one-hot encoding of the action ID (dimension D=18) without additional projection layers [38].

The fused features and action embeddings are concatenated to form a composite vector of dimension 146 (128+18). This vector is fed into the Classifier, which consists of a fully connected layer (hidden size 128), a ReLU activation, a Dropout layer (rate = 0.3) to prevent overfitting, and a final linear output layer. The output dimension is 6, corresponding to the six discrete recovery states (Score 0–2 for both Extension and Flexion tasks).

### 2.5. Classification Task Formulation

To achieve automated and objective assessment, we formulated the upper-limb motor function evaluation as a six-class classification task, aligning with FMA-UE protocols for elbow flexion and extension. The FMA-UE sub-scores are discretized into three levels: 0 (severe impairment), 1 (moderate impairment), and 2 (mild impairment). To map these attributes into a unified label space, we employed a parity-based encoding scheme defined by:

y=2s+p

where s∈{0,1,2} denotes the FMA-UE score and p∈{0,1} denotes the action parity (P=0 for extension, P=1 for flexion). This encoding yields six distinct outcome categories: Classes 0 and 1 correspond to Score 0 (Severe), 2 and 3 to Score 1 (Moderate), and 4 and 5 to Score 2 (Mild). Specifically, the incorporation of 18 distinct action variations is designed to characterize the mapping of flexion and extension motor functions across varying ranges of motion (ROM). Consequently, the AMWFNet simultaneously determines the patient’s functional capability with enhanced precision across diverse task contexts.

### 2.6. Model Training and Evaluation

#### 2.6.1. Data Balancing and Subject-Wise Partitioning Strategy

To address the significant class imbalance observed in the raw dataset—where the Score 2 category (normal function) predominantly outnumbers Score 0 and Score 1 samples (as detailed in Table 2)—and to ensure the model’s generalization capability to unseen subjects, we implemented a rigorous data curation protocol.

Our experimental design follows a “Subject-wise Split-then-Augment” strategy (in Table 3), structured as follows:

1. Subject-wise Partitioning: We implemented a strict subject-wise partitioning with a 60%:20%:20% split (corresponding to 24 training, 8 validation and 8 test subjects) to prevent the model from overfitting to subject-specific idiosyncrasies, such as skin impedance or unique motor habits. This isolation of test subjects is crucial for eliminating data leakage and simulating the diagnosis of new patients. Consequently, our evaluation metrics reflect the model’s true generalization capability and robustness in a realistic clinical setting [39].
2. Training Set Balancing and Augmentation: Following the data partitioning, data augmentation was applied to the training set with the primary objective of balancing the sample distribution across the three impairment levels (Score 0, 1, 2) and enhancing model robustness. We employed specific transformation techniques to generate synthetic samples for under-represented classes, ensuring an equitable representation of functional states. The augmentation protocol included three methods [40]: (1) Amplitude Scaling, where signals were randomly adjusted by a factor α∈[0.9,1.1] to simulate inter-subject variations in muscle activation intensity and sensor gain; (2) Time Warping, utilized to model the temporal elasticity and inconsistent movement speeds characteristic of stroke survivors by stretching or compressing the time-axis by ±10% (scaling factor ∈[0.9,1.1]). Crucially, the same warping factor was applied synchronously to sEMG, velocity, and acceleration signals to preserve temporal alignment across modalities; and (3) Gaussian Noise Injection, introduced to improve robustness against sensor interference by injecting independent Gaussian white noise ($N\left(0,\;\sigma^{2}\right)$). We adopted a modality-specific noise intensity to maintain physical plausibility: a higher noise level ($\sigma_{emg}=0.05$) was applied to sEMG signals to simulate electronic noise, while a conservative level ($\sigma_{kine}=0.005$) was used for kinematic signals (velocity and acceleration) to mimic sensor measurement uncertainty without introducing unrealistic jitter. Furthermore, a Physical Consistency Constraint was enforced: these augmentations were strictly applied to the sEMG, velocity, and acceleration profiles, while Jerk was explicitly excluded from direct augmentation to prevent the generation of physically implausible artifacts common in higher-order derivatives. Consequently, the validation and test sets remained unaugmented to reflect real-world performance.
3. Evaluation Integrity: The validation and test sets were maintained in their raw, unaugmented state. Preserving the original distribution in these sets ensures that the evaluation metrics accurately reflect the model’s diagnostic performance on real-world, unseen patient data.

#### 2.6.2. Two-Stage Training Protocol

We utilized a stage-wise transfer learning approach to stabilize convergence:

Stage 1: Initialization on Robot-Guided Passive Training (Warm-up). The model is first trained on the training partition of the balanced Robot-Guided Passive Training dataset (comprising approximately 8399 samples). This data is characterized by consistent kinematic trajectories and minimal voluntary sEMG. It acts as a domain-specific regularizer, allowing the encoder to efficiently converge on fundamental signal structures. We trained for 100 epochs with a learning rate of $1\times 10^{-3}$.

Stage 2: Fine-tuning on Active Voluntary Training. Subsequently, the pre-trained weights are fine-tuned on the Active Voluntary Training dataset (approx. 28,692 samples). Given the complex pathological patterns and higher variability in this dataset, we reduced the learning rate to $5\times 10^{-4}$. This refinement phase adjusts the decision boundaries for the target stroke population, accelerating convergence while preventing the model from becoming trapped in local minima induced by signal noise.

#### 2.6.3. Implementation Details

The proposed framework was implemented using PyTorch (version 2.2.2) with CUDA 11.8 support on a workstation equipped with an Intel Xeon Silver 4314 CPU and a single NVIDIA GeForce RTX 3090 GPU (24 GB VRAM). The model parameters were optimized using the AdamW optimizer with a weight decay of $1\times 10^{-4}$ to mitigate overfitting. We minimized the Cross-Entropy Loss using a batch size of 32. To enhance training stability and efficiency, we utilized Automatic Mixed Precision (AMP) and applied gradient clipping with a maximum norm of 1.0. An early stopping mechanism was employed to halt training if the validation loss did not improve for 10 consecutive epochs.

#### 2.6.4. Evaluation Metrics

To comprehensively assess model performance, we utilized a multi-level evaluation protocol. Quantitative assessment relied on overall Accuracy, as well as Precision, Recall, and F1-Score. To account for potential class imbalances, these metrics were computed as macro-averages derived from the confusion matrix [41]. Qualitative analysis focused on model interpretability and clinical relevance, specifically through: (1) Attention Weight Visualization to generate muscle importance heatmaps; (2) Gating Weight Distribution to analyze the contribution of each modality; (3) Confusion Matrix Analysis to identify specific class misclassifications.

### 2.7. Baseline Machine Learning Models

To benchmark the AMWFNet, we compared it against both traditional machine learning models (SVM, RF) trained on hand-crafted features and a raw-signal deep learning baseline (1D-CNN) [9,42].

For the traditional models, a 112-dimensional vector was constructed, comprising: (1) 64 sEMG dimensions, derived from 8 metrics per muscle including time-domain (RMS, MAV, VAR, WL, ZC, SSC) and frequency-domain (MDF, MPF) indicators [43]; (2) 30 kinematic dimensions, calculated using 10 statistical measures per channel (mean, std, max, min, median, IQR, RMS, peak-to-peak, jerk index, spectral arc length) [44]; and (3) an 18-dimensional action encoding.

The SVM utilized an RBF kernel (C=10.0, γ={‘scale’}), and the RF used 300 estimators (min_samples_leaf = 2). Additionally, we implemented a dual-stream 1D-CNN that processes raw time-series data directly [45]. This baseline utilizes a parallel architecture similar to AMWFNet but replaces 2D CWT encoders with 1D convolutional layers, serving to validate the necessity of time-frequency transformation.

All baselines applied class-balanced weighting and were trained to predict FMA-UE Elbow Flexion and Extension sub-scores. We strictly adhered to the subject-wise split (6:2:2) strategy. Performance was evaluated on the held-out test set using the evaluation metrics defined in Section 2.6.4.

## 3. Results

### 3.1. Overall Classification Performance

The comparative results are presented in Table 4. The proposed AMWFNet achieved superior performance with an accuracy of 94.68% and an F1-score of 91.75%.

In contrast, traditional models (RF, SVM) relying on manual feature engineering plateaued at significantly lower accuracies (~85.5%), highlighting the insufficiency of hand-crafted features in capturing complex stroke kinematics. Notably, while the deep learning baseline (1D-CNN) improved accuracy to 91.21% by leveraging raw signal feature learning, AMWFNet still maintained a 3.47% lead. This performance margin validates the efficacy of transforming 1D signals into 2D time-frequency scalograms (CWT), which captures richer spatio-temporal dynamics than direct 1D processing.

### 3.2. Per-Class Assessment Analysis

**Figure 6:**
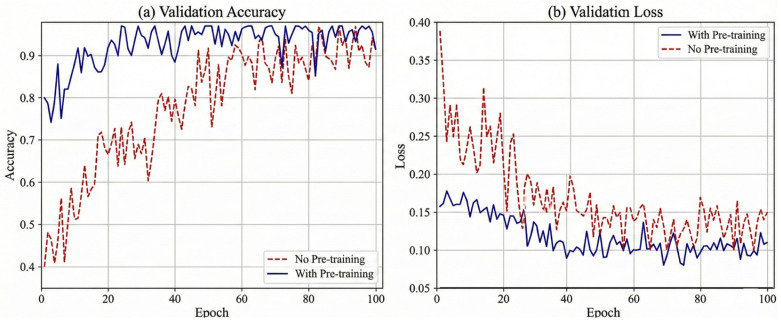
Impact of pre-training strategy on training dynamics.

Figure 6 demonstrates that the two-stage protocol significantly improves convergence speed, performance, and stability compared to the baseline.

As shown in Figure 6a, the pre-trained model (blue line) exhibits a “jump-start” effect, achieving an initial accuracy of 79.65% compared to ≈40.21% for the baseline. This advantage stems from learned prior knowledge: pre-training initializes the gating weights to an optimized ratio. Unlike the baseline’s naïve 0.5:0.5 split, this optimized ratio prioritizes robust kinematic features, accelerating early-stage learning. Consequently, the pre-trained model converges faster and attains a higher peak accuracy (>96%), indicating that prior learning helps the encoder navigate the loss landscape toward a better optimum. Figure 6b highlights the stability benefits. The baseline (red line) suffers from significant loss fluctuations, likely due to sensitivity to sEMG variability under equal weighting. In contrast, the pre-trained model shows smooth, monotonic convergence. By maintaining a higher weight for kinematics (0.744), the model effectively mitigates sEMG artifacts and prevents the oscillations observed in the baseline.

### 3.3. Ablation Studies

To verify the contribution of individual components within the AMWFNet architecture, we conducted comprehensive ablation studies. The performance comparison of different configurations is summarized in Table 4.

#### 3.3.1. Impact of Multimodal Fusion

We first evaluated the necessity of fusing physiological and kinematic data. As shown in Table 5, using sEMG alone yielded an accuracy of 86.19%, while using kinematics alone resulted in 91.64%. While kinematics serves as a strong predictor for FMA sub-scores (which are movement-based), the Full Model achieved the highest accuracy of 94.68%. This represents an improvement of 3.04% over the best single modality, confirming that sEMG (reflecting neural drive) and kinematics (reflecting execution) provide complementary information essential for precise assessment.

#### 3.3.2. Effectiveness of Modality Gating

**Figure 7:**
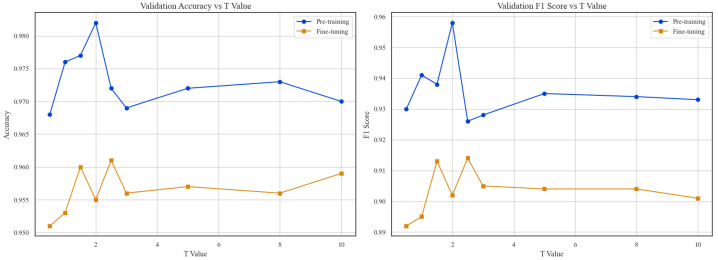
Hyperparameter sensitivity analysis of the gating temperature (τ).

We investigated the sensitivity of the gating mechanism to τ∈{0.5, 1.0, 1.5, 2.0, 2.5, 3.0, 5.0, 8.0, 10.0}. As shown in Figure 7, performance exhibits a non-monotonic trend, peaking at τ=2.5 (Accuracy: 96.12%, F1: 91.53%).

τ regulates the entropy of the modality selection. Lower values (e.g., τ=1.5) force a “winner-takes-all” behavior, aggressively suppressing complementary sEMG cues and degrading performance. Conversely, higher values (τ≥5) approximate a simple average fusion, failing to filter noise from unreliable channels. The optimal setting (τ=2.5) strikes a critical balance: it is sharp enough to prioritize high-fidelity kinematics yet soft enough to integrate essential fine-grained neuromuscular details.

**Figure 8:**
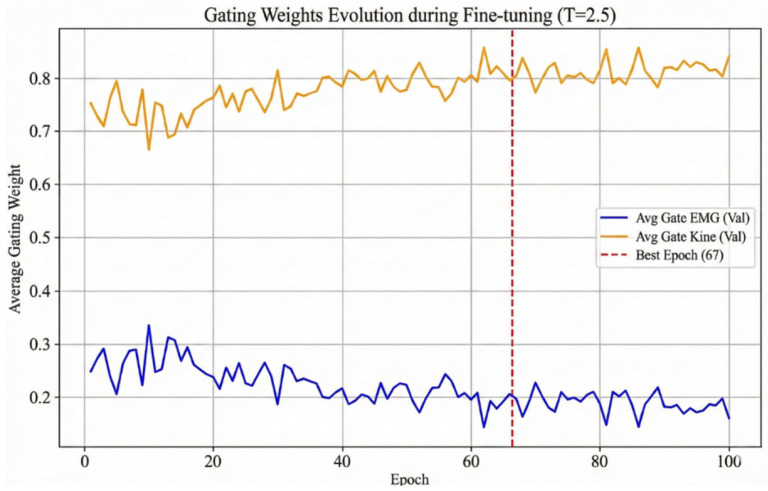
Dynamic adaptation of modality importance weights.

Comparison between fusion strategies demonstrated the clear superiority of our Modality Gating mechanism. The proposed gating approach outperformed the naive feature concatenation strategy by 2.51% in accuracy. To understand this improvement, we visualized the evolution of gating weights during the fine-tuning stage (Figure 8).

As training converged (Best Epoch: 67), the network learned a stable weight distribution of 0.804 for Kinematics and 0.196 for sEMG, resulting in an approximate 4:1 ratio. This distribution aligns with clinical intuition: kinematic trajectories provide the primary, stable descriptor of motor execution, while sEMG offers critical supplementary information regarding neural drive. The gating mechanism allows the model to prioritize the high-fidelity kinematic signal while retaining essential neuromuscular cues that purely kinematic models miss.

#### 3.3.3. Contribution of Action Embedding

**Figure 9:**
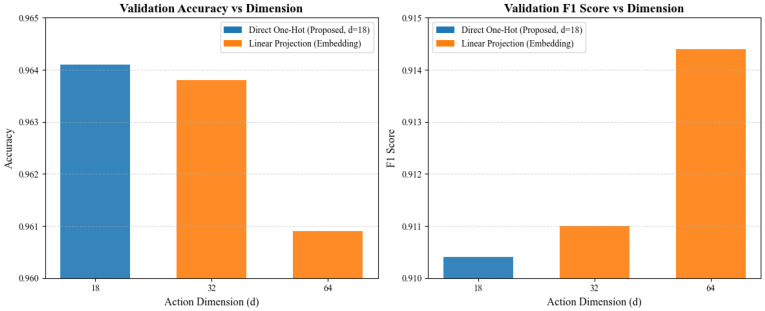
Sensitivity analysis of the action one-hot embedding dimension.

To determine the optimal representation for action encoding, we conducted an ablation study comparing the raw one-hot encoding (D=18) against higher-dimensional embeddings (D=32, 64) projected via linear layers. The results are presented in Figure 9.

We observed that increasing the embedding dimension did not yield a consistent performance improvement. Specifically, the model achieved the highest validation accuracy (~96.41%) with the 18-dimensional one-hot vector. While projecting to 64 dimensions resulted in a marginal increase in the F1 score (+0.4%), it led to a decrease in overall accuracy (−0.3%). This suggests that the higher-dimensional projections introduced additional model complexity without capturing more discriminative features. Consequently, to prioritize parameter efficiency and model simplicity, we selected the 18-dimensional one-hot encoding for the final architecture.

### 3.4. Model Interpretability and Visualization

**Figure 10:**
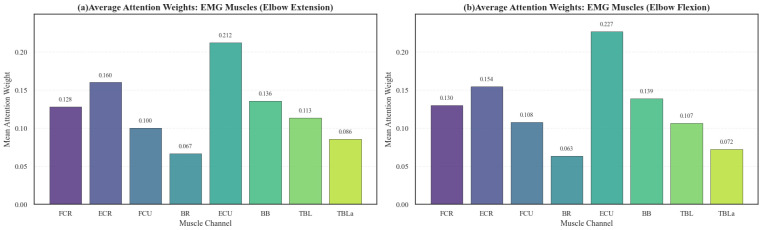
Average attention weights assigned to sEMG channels across three planar reaching tasks.

The Channel-wise Attention Pooling module elucidates the model’s decision-making by quantifying muscle channel contributions. Figure 10 reveals two primary physiological insights based on attention weight distribution:

Unexpectedly, the model assigned higher importance to distal forearm muscles than to proximal prime movers, with the ECU, ECR, and FCR receiving the highest weights. Specifically, the ECU peaked at 0.212 (Extension) and 0.227 (Flexion), reflecting the compensatory forearm strategies common in stroke survivors. Thus, wrist stabilization associated with pathological synergies emerged as a critical discriminative feature.

**Figure 11:**
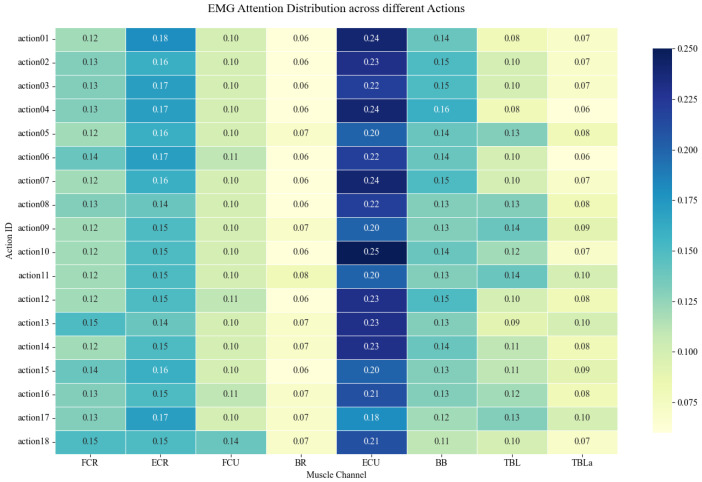
Visualization of pathological coupling and compensatory patterns via sEMG attention weights.

Meanwhile, the proximal Biceps (BB) and Triceps (TBL) maintained stable weights (~0.14 and ~0.11), confirming that while distal muscles reveal compensatory patterns, proximal recruitment remains essential for generating gross torque during elbow flexion and extension. This pattern is corroborated by the action-wise heatmap (Figure 11), which shows a continuous high-attention band across the distal forearm across all 18 actions. This demonstrates that AMWFNet captures task-invariant pathological coupling, validating distal synergies as robust biomarkers for functional assessment.

**Figure 12:**
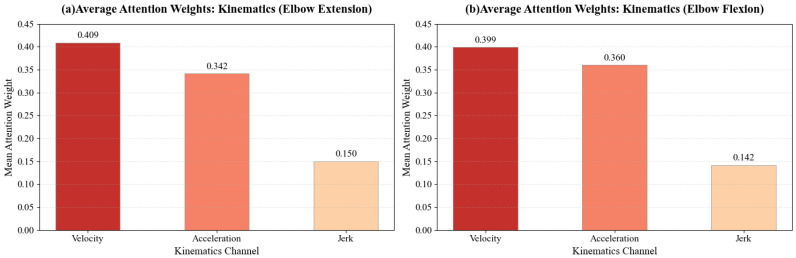
Global comparison of average attention weights across kinematic features.

Complementing the physiological analysis, the Attention mechanism elucidates which movement characteristics define motor recovery levels. The distribution of kinematic attention weights (Figure 12) reveals a distinct hierarchical prioritization:

Regarding kinematic features, the model exhibited a clear hierarchical attention distribution. Velocity emerged as the dominant factor, achieving the highest weights for both Elbow Extension (0.409) and Flexion (0.399). Acceleration played a secondary role (weights ≈ 0.34–0.36), reflecting a focus on movement dynamics. In contrast, Jerk consistently received the lowest importance (≈0.14–0.15), indicating that for this classification task, the model prioritizes gross motor trajectory and force control features over movement smoothness metrics.

**Figure 13:**
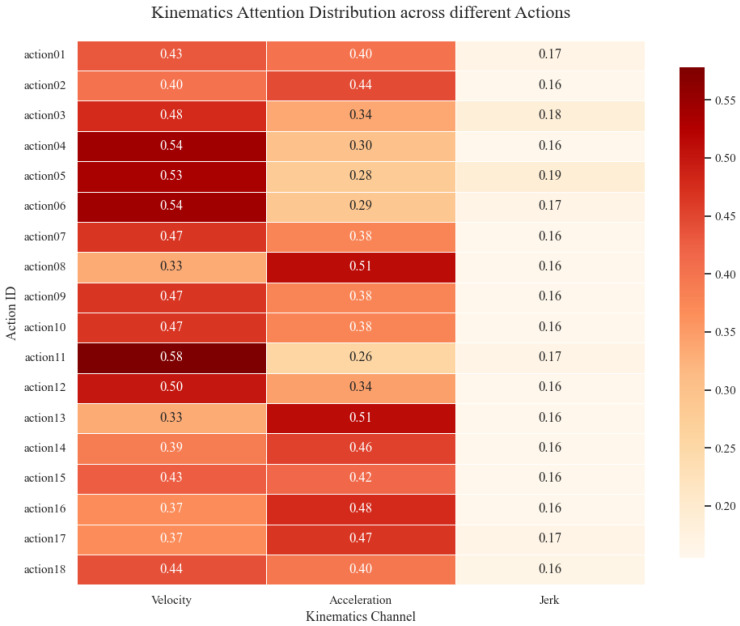
Distribution of attention weights across kinematic channels (Velocity, Acceleration, Jerk) for the 18 reaching tasks.

The attention distribution remained consistent between flexion and extension but varied significantly by spatial orientation. We observed an inverse relationship between movement angle and feature prioritization: as the reaching angle increased (45° → 135°), the model’s attention shifted from Velocity to Acceleration. Meanwhile, Jerk weights remained stable across all 18 actions (Figure 13).

**Figure 14:**
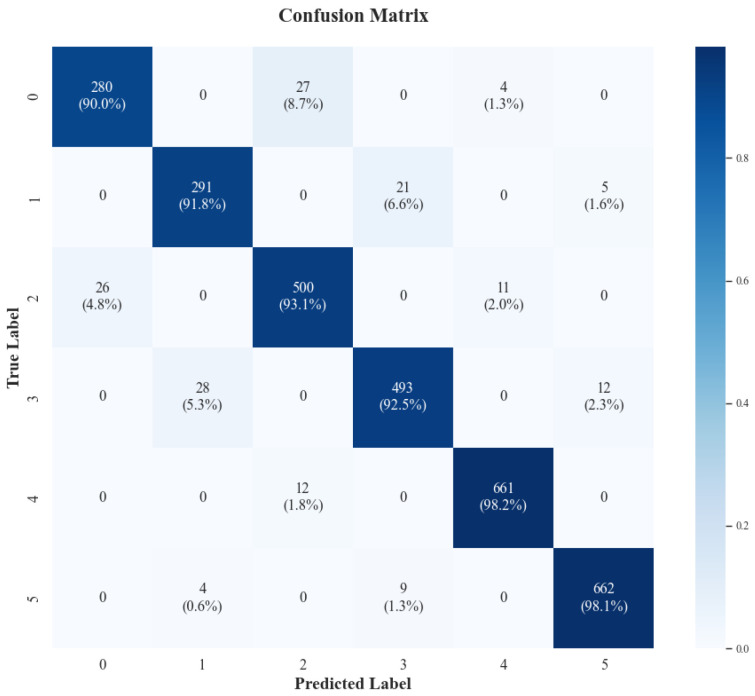
Visualization of classification performance via Confusion Matrix on the test set.

Figure 14 illustrates the confusion matrix of the fine-tuned model on the six target classes. The high diagonal density confirms the model’s overall robustness, particularly in distinguishing specific movement patterns (Extension and Flexion) across different clinical impairment levels (Score 0, Score 1, and Score 2).

To validate that the model’s accuracy is not driven solely by healthy data, we conducted a stratified analysis restricted to the stroke cohort. The results confirm robust discriminative power between severe (Score 0) and moderate (Score 1) impairment: the model achieved a recall of 90.0% for Score 0 extension and 91.8% for Score 0 flexion. Misclassifications were predominantly clustered between these adjacent levels (e.g., 8.7% of Score 0 samples misclassified as Score 1), reflecting the clinical subtlety between impairment grades rather than random error.

Simultaneously, the model exhibited near-perfect isolation of healthy patterns (Score 2), achieving >98% recall for both extension and flexion tasks. Crucially, the system demonstrated high clinical safety by minimizing “cross-level” errors. Only 1.3% of severe impairment samples (Score 0) were incorrectly predicted as normal (Score 2), ensuring that the system minimizes the risk of gross overestimation when prescribing rehabilitation intensities.

## 4. Discussion

### 4.1. Multimodal Fusion Effectiveness

The ablation study confirms that sEMG and kinematic data are highly complementary. sEMG directly reflects neural intention and muscle physiological state, while kinematics quantify the resulting motion execution. Our modality gating mechanism provides a significant advantage over simple concatenation, yielding a 1.9% accuracy gain. The temperature-scaled softmax (τ=2.5) enables smooth, adaptive weighting that prevents modality collapse.

Crucially, this mechanism allows the model to dynamically determine the optimal ratio of EMG to kinematic data based on signal reliability. This finding suggests a promising avenue for future system optimization: by leveraging the strong predictive power of kinematics, high-accuracy assessment may be achievable with fewer, strategically selected EMG channels. This reduction in hardware complexity would significantly facilitate “assessment-training integration,” making the system easier to deploy for human–robot collaborative rehabilitation in clinical settings.

### 4.2. CWT Feature Representation Advantages

Compared to traditional hand-crafted features (e.g., RMS, MNF), CWT scalograms capture richer spatio-temporal dynamics by preserving non-stationary signal characteristics. However, the most critical advantage of this image-based approach lies in its ability to seamlessly align heterogeneous sensor streams.

In multimodal rehabilitation systems, synchronizing signals with disparate sampling rates (e.g., 1 kHz for sEMG vs. 500 Hz for robotics) typically necessitates aggressive interpolation or downsampling, which can distort signal fidelity and introduce aliasing artifacts. Our framework circumvents this bottleneck by converting 1D time-series into a unified 2D time-frequency spatial domain. Regardless of the native sampling rate, the CWT projects all modalities into standardized scalograms (e.g., 128×128 resolution). This inherently solves the alignment challenge, allowing the CNN encoder to learn cross-modal correlations directly from the aligned images without the information loss associated with temporal resampling.

### 4.3. Interpretability and Clinical Insights

The AttentionPool module provides a transparent, ‘white-box’ perspective on the assessment logic, facilitating the post hoc identification of pathological markers such as compensatory strategies (elevated synergist activity) and spasticity (antagonist co-activation). Crucially, our analysis revealed that a specific subset of muscles—namely the FCR, ECR, ECU, BB, and TBL—consistently exhibited the highest attention weights, identifying them as the most discriminative channels for functional classification. This finding offers a tangible clinical insight: the current 8-channel sEMG setup could be optimized by pruning redundant sensors to focus on these high-sensitivity muscles. Such a reduction would significantly enhance deployment efficiency and reduce patient setup time without compromising assessment accuracy.

Regarding kinematics, the dominance of Velocity weights (Figure 12) aligns with clinical reality, where movement speed and amplitude are primary indicators of volitional motor control recovery in the FMA-UE scale. Furthermore, the adaptive shift from Velocity to Acceleration observed in larger-angle tasks (Figure 13) reflects biomechanical constraints: wider angles impose greater stability demands, requiring sustained force application (Acceleration), whereas shorter, ballistic movements are primarily characterized by speed. This confirms the model’s ability to dynamically adjust its evaluation focus based on task-specific mechanical requirements.

### 4.4. Six-Class Classification Assessment Paradigm

We established a refined classification paradigm that treats assessment as a joint prediction of functional ability (FMA-UE Elbow Flexion and Extension sub-scores) and movement context (Action ID). By mapping standardized, planar robotic movements to specific clinical scores, we move beyond binary “healthy vs. patient” classification. This approach demonstrates that quantitative data from standard planar reaching tasks can effectively proxy the complex, multi-joint evaluation criteria of the Fugl-Meyer scale, providing a standardized, objective metric that bridges the gap between robotic training data and clinical decision-making. Specifically, by prioritizing the precise classification of elbow sub-items, this work establishes a foundational step toward a fully automated, comprehensive FMA assessment system.

### 4.5. Limitations and Future Work

The current study is limited to a single-center cohort of 40 participants. We are currently organizing multi-center validation with larger datasets to ensure generalizability.

Currently, our framework focuses on quantifying two specific sub-items (Elbow Flexion and Extension) within a 2D workspace. While this serves as a foundational study for assessment-training integration, we acknowledge the need for comprehensive 3D evaluation. Future work will involve extending the protocol to 3D robotic systems and integrating additional sensor modalities (e.g., IMUs or motion capture) to assess the remaining 31 FMA-UE items, ultimately achieving a holistic automated scoring system.

The proposed AMWFNet is designed to be lightweight for potential edge deployment. The total number of trainable parameters is approximately 1.27 million (M), resulting in a physical model size of roughly 4.84 MB. This compact architecture contributes to a fast inference speed (~922 ms per trial on CPU). To further facilitate integration into rehabilitation robot controllers, we plan to implement model quantization and TensorRT optimization, enabling real-time, low-latency feedback during training sessions.

Building on our interpretability findings, we will investigate channel pruning strategies to remove redundant EMG sensors (e.g., reducing from 8 to 5 channels), thereby enhancing the clinical usability of the system.

## 5. Conclusions

This study presented the Action-Aware Multimodal Wavelet Fusion Network (AMWFNet), a novel deep learning framework designed to automate the quantitative assessment of upper-limb motor function in post-stroke rehabilitation. By transforming heterogeneous sEMG and kinematic signals into unified CWT scalograms and employing a dynamic modality gating mechanism, the framework effectively synthesizes neural intent with movement execution, addressing the limitations of single-modality approaches.

Our extensive validation on 40 participants demonstrated that AMWFNet achieves a state-of-the-art accuracy of 94.68% in classifying six-level functional states aligned with FMA-UE scores. Beyond high classification performance, the interpretability analysis provided crucial physiological insights, identifying distal synergies (ECU, ECR) and key muscle groups (FCR, BB, TBL) as high-sensitivity biomarkers for motor recovery. This finding supports the potential for optimizing sensor layouts to improve clinical usability.

The framework offers three key advantages: (1) CWT-based representation handles sensor heterogeneity while preserving signal fidelity; (2) action-aware embedding explicitly encodes task-specific context across 18 standardized tasks, significantly improving accuracy (94.68% vs. 54.46% without embedding) and accelerating convergence; (3) learnable modality gating dynamically weights modalities; (4) lightweight architecture (1.27 M parameters, 4.84 MB, ~922 ms inference) suits edge deployment in rehabilitation robots. By automating the mapping from sensor data to FMA-UE scores, AMWFNet enables real-time assessment-training integration, overcoming the subjectivity and inefficiency of manual clinical scales.

Furthermore, the model’s lightweight architecture—comprising approximately 1.27 million parameters with a storage footprint of 4.84 MB—enables fast inference (~922 ms), making it highly suitable for deployment on edge computing devices within rehabilitation robots. Collectively, this work advances the paradigm of “assessment-training integration,” offering a robust, objective, and real-time solution to overcome the subjectivity of traditional clinical scales and facilitating personalized, human–robot collaborative therapy.

## Figures and Tables

**Table 1:** Demographic and clinical characteristics of the participants.

Variables	Healthy (*n* = 20) Mean ± SD	Post-Stroke (*n* = 20) Mean ± SD
Age (years)	54.1 ± 13.6	49.0 ± 11.3
Sex (Male/Female)	10/10	10/10
Height (cm)	173.9 ± 9.3	169.7 ± 8.9
Weight (kg)	68.8 ± 10.2	72.1 ± 9.9
Body Mass Index (kg/m^2^)	22.9 ± 3.0	24.0 ± 3.0
Affected side (Left/Right)	-	5/15
FMA-UE Total Score (0–66)	-	35.2 ± 8.7
FMA-UE Elbow Flexion (0/1/2)	-/-/20	8/12/0
FMA-UE Elbow Extension (0/1/2)	-/-/20	8/12/0

**Table 2:** Distribution of samples across impairment levels (FMA-UE Scores) utilized in the two-stage training protocol.

Impairment Level	Stage 1: Robot-Guided Passive Training	Stage 2: Active Voluntary Training	Total Samples
Score 0 (Severe)	2258	3042	5300
Score 1 (Moderate)	2990	5695	8685
Score 2 (Normal)	6951	10,955	17,906
Total	12,199	19,692	31,892

**Table 3:** Detailed Data Flow and Sample Counts throughout the Two-Stage Training.

Stage	Total Raw Dataset (from Table 2)	Data Partitioning (Train/Val/Test)	Raw Training Samples	Augmentation Strategy	Final Effective Training Samples (Used in Model)
Stage 1 (Passive)	12,199	Subject-wise (60%:20%:20%)	~7319	Balancing and Noise Injection	8399
Stage 2 (Active)	19,692	Subject-wise (60%:20%:20%)	~11,815	Class Balancing and Augmentation	28,692

**Table 4:** Performance comparison between the proposed AMWFNet and baseline models on the six-class classification task.

Model	Feature Type	Accuracy	Precision	Recall	F1-Score
AMWFNet	CWT + Action Embedding	94.68%	91.99%	91.55%	91.75%
Random Forest (RF)	Manual (Time/Freq)	85.51%	84.32%	85.26%	84.71%
SVM	Manual (Time/Freq)	85.30%	85.16%	85.56%	85.18%
1D-CNN	Raw Signal	91.21%	90.18%	90.14%	90.09%

**Table 5:** Ablation study results comparing different model configurations on the six-class classification task.

Method	Accuracy	Precision	Recall	F1-Score
sEMG Only	86.19%	74.41%	74.27%	76.04%
Kinematics Only	91.64%	87.44%	84.64%	85.96%
sEMG + Kinematics (no Action Emb.)	54.46%	52.05%	53.18%	51.01%
sEMG + Kinematics + Action Emb. (no Gating)	92.17%	90.74%	90.06%	90.77%
Full Model (Proposed)	94.68%	91.99%	91.55%	91.75%

## Data Availability

The dataset is not publicly available due to patient privacy regulations but can be accessed upon reasonable request to the corresponding author, subject to IRB approval.

## Abbreviations

The following abbreviations are used in this manuscript:

1D-CNN	One-Dimensional Convolutional Neural Network
AMP	Automatic Mixed Precision
AMWFNet	Action-Aware Multimodal Wavelet Fusion Network
ARAT	Action Research Arm Test
BB	Biceps Brachii (Muscle)
BR	Brachioradialis (Muscle)
CNN	Convolutional Neural Network
CPU	Central Processing Unit
CWT	Continuous Wavelet Transform
ECR	Extensor Carpi Radialis (Muscle)
ECU	Extensor Carpi Ulnaris (Muscle)
FCR	Flexor Carpi Radialis (Muscle)
FCU	Flexor Carpi Ulnaris (Muscle)
FMA-UE	Fugl-Meyer Assessment for Upper-Limb (or Upper-Extremity)
GPU	Graphics Processing Unit
IQR	Interquartile Range
IRB	Institutional Review Board
MAS	Modified Ashworth Scale
MAV	Mean Absolute Value
MDF	Median Frequency
MLP	Multilayer Perceptron
MPF	Mean Power Frequency
ReLU	Rectified Linear Unit
RF	Random Forest
RMS	Root Mean Square
ROM	Ranges of Motion
sEMG	Surface Electromyography
SSC	Slope Sign Change
SVM	Support Vector Machine
TBL	Triceps Brachii Long Head (Muscle)
TBLa	Triceps Brachii Lateral Head (Muscle)
VAR	Variance
VRAM	Video Random Access Memory
WL	Waveform Length
WMFT	Wolf Motor Function Test
ZC	Zero Crossing
